# When the Body Meets Space: The Contribution of Interoception and Emotional Object-Location Memory as Predictors of Health-Related Quality of Life in Aging

**DOI:** 10.3390/healthcare14203414

**Published:** 2026-10-13

**Authors:** Sabrina Iuliano, Scila Nunziata, Maria Rosaria Pasciucco, Francesca Ferri, Marcello Costantini, Gennaro Ruggiero

**Affiliations:** 1 Department of Psychology, University of Campania “Luigi Vanvitelli”, Viale Ellittico 31, 81100 Caserta, Italy; sabrina.iuliano@unicampania.it (S.I.); scila.nunziata@unicampania.it (S.N.); 2 Department of Neuroscience, Imaging and Clinical Sciences, “Gabriele d’Annunzio” University of Chieti and Pescara, Via dei Vestini, 33, 66100 Chieti, Italy; mariarosaria.pasciucco@unich.it (M.R.P.); francesca.ferri@unich.it (F.F.); marcello.costantini@unich.it (M.C.); 3 Institute for Advanced Biomedical Technologies, “Gabriele d’Annunzio” University of Chieti and Pescara, Via dei Vestini, 33, 66100 Chieti, Italy

**Keywords:** aging, health-related quality of life, functional ability, interoceptive awareness, spatial memory, emotional processing, object-location memory

## Abstract

**Highlights:**

- Q1: What predicts health-related quality of life across adulthood?
- A1: The integration of interoceptive regulation and emotional object-location memory contributes to predicting health-related quality of life.
- Q2: Does emotional object-location memory relate differently to health-related quality of life in younger and older adults?
- A2: Yes, its contribution differs between age groups, suggesting distinct patterns of adaptation.
- Q3: What role does body–environment integration play in adaptive functioning?
- A3: Body–environment integration may represent an important mechanism supporting adaptive functioning across adulthood.
- Q4: What are the potential implications for interventions?
- A4: Interventions targeting interoceptive regulation and emotional-spatial processing may offer a promising avenue for supporting health-related quality of life.

**Abstract:**

Background/Objectives: Adaptive functioning involves the integration of bodily states with emotional information from the external environment. Interoceptive functioning and spatial cognition may contribute to this integration, but their joint associations with health-related quality of life (HRQoL) across adulthood remain insufficiently understood. Methods: We examined associations between multidimensional interoceptive functioning, emotional object-location memory, and HRQoL in younger adults (*n* = 43) and older adults (*n* = 38). Participants completed an immersive virtual-reality Object Location Memory task assessing object recognition (What), spatial location memory (Where), and object-location binding (What + Where) for positive, negative, and neutral stimuli. Interoceptive functioning was assessed using the Multidimensional Assessment of Interoceptive Awareness (MAIA) and the Body Perception Questionnaire (BPQ), and HRQoL was assessed using the SF-36. For each SF-36 domain and age group, interoceptive predictors were screened using bootstrap-based LASSO selection and subsequently entered into multiple regression linear models together with the prespecified spatial-memory predictors. Model robustness was further examined using bootstrap, leave-one-out cross-validation, and permutation testing. Results: The regression analyses identified associations between interoceptive functioning, emotional object-location memory, and HRQoL. In older adults, selected dimensions of interoceptive functioning, particularly those related to attention regulation, adaptive engagement with bodily sensations, and confidence in bodily signals, were associated with several HRQoL domains. Emotional object-location memory also showed domain-specific associations, with positive- and negative-valence measures contributing differently across HRQoL outcomes. In younger adults, selected associations were also observed between interoceptive functioning, emotional object-location memory, and HRQoL, including associations involving neutral and positive object-location information. Conclusions: The findings provide exploratory evidence that specific dimensions of interoceptive functioning and emotionally modulated object-location memory are associated with selected aspects of self-reported HRQoL, with distinct patterns of associations emerging in younger and older adults.

## 1. Introduction

Population aging represents one of the major public health challenges of the coming decades, requiring a better understanding of the mechanisms that support autonomy, independence, and health-related quality of life (HRQoL) in later adulthood. According to the World Health Organization [1,2], the number of adults aged 60 years and older is expected to reach approximately 2.1 billion by 2050, with a consequent increase in healthcare and long-term care demands [1,2]. Within the WHO framework of Healthy Ageing, functional ability is defined as the capacity to be and to do what individuals value, emerging from the dynamic interaction between intrinsic capacities and the environments in which people live. Identifying the mechanisms supporting functional ability is therefore essential for understanding successful aging and developing strategies aimed at preserving autonomy and quality of life.

Adaptive functioning requires continuous adjustment between internal bodily states and external environmental demands. Within embodied cognition frameworks, cognition is not considered an isolated process independent from the body and the environment; rather, adaptive behavior emerges from the integration of information concerning the external world with information concerning the individual’s physiological state [3,4,5,6]. Exteroceptive systems provide representations of objects, events, and spatial relationships, whereas interoception monitors internal bodily signals involved in bodily awareness, emotional experience, self-regulation, and adaptive behavior [7,8]. The interaction between these systems allows individuals to construct coherent embodied representations that guide perception, memory, decision-making, and goal-directed actions [9].

A cognitive domain in which body–environment integration becomes particularly evident is object-location memory. Successful interaction with everyday environments requires individuals not only to recognize relevant objects but also to encode their spatial position and establish associations between object identity and spatial context. Object-location memory comprises three partially dissociable components: object recognition (what), spatial location processing (where), and object-location binding (what + where) [10]. While object recognition and spatial processing rely on distributed cortical networks, object-location binding depends primarily on hippocampal and medial temporal lobe structures supporting associative memory processes [11,12,13]. Importantly, object-location representations are not purely visuospatial but are continuously shaped by the interaction between the observer and the surrounding environment through body-centred reference systems [9]. Therefore, object-location memory represents an ecologically relevant model for investigating how external spatial information is integrated with embodied representations to support adaptive behavior.

Healthy aging provides a particularly informative framework for investigating these mechanisms because it is accompanied by changes affecting both spatial memory and bodily processing. Object-location memory is especially vulnerable to aging because hippocampal-dependent associative binding processes decline earlier and more markedly than simpler recognition abilities [14,15,16]. Reduced efficiency in binding object identity and spatial context may affect the ability to construct coherent representations of the surrounding environment, potentially influencing everyday autonomy and independent functioning.

At the same time, aging is associated with changes in interoceptive processing. Previous evidence indicates age-related alterations in both interoceptive accuracy and awareness [17,18,19]. However, aging is increasingly conceptualized as a dynamic and adaptive process rather than as a simple decline of bodily and cognitive abilities. Individual differences, compensatory mechanisms, and changes in body–brain coupling may contribute to maintaining successful adaptation despite age-related modifications [20,21,22]. Within this perspective, interoception is considered a multidimensional construct that includes not only the detection of bodily sensations but also attention regulation, emotional interpretation, self-regulation, and confidence in bodily signals [23,24]. These processes rely on large-scale networks involved in bodily monitoring and salience detection, particularly the anterior insula and anterior cingulate cortex within the Salience Network [25,26]. Consequently, age-related differences in interoceptive functioning may influence how bodily states are integrated with environmental representations and how individuals adapt their behavior to everyday demands.

Importantly, body–environment integration is not emotionally neutral. Environmental information differs in its behavioral relevance, and emotionally salient stimuli receive prioritized processing because they signal opportunities, threats, or personally meaningful events [27]. Neurocognitive models have demonstrated that emotional salience influences memory formation through interactions between the amygdala, hippocampus, and distributed cortical systems, modulating attention, encoding, and retrieval processes [27,28,29,30]. Because emotionally relevant events also elicit autonomic and visceral responses continuously monitored through interoceptive processes, emotional memory emerges from the integration of sensory, affective, and bodily information [8,31,32]. Within object-location memory, emotional valence may therefore influence not only object recognition and spatial location processing but also the associative binding between objects and their spatial context.

The interaction between emotion and memory undergoes important changes across adulthood. According to Socioemotional Selectivity Theory [33], advancing age is associated with increased prioritization of emotionally meaningful goals, often resulting in enhanced processing of positive relative to negative information, a phenomenon known as the positivity effect [33,34,35]. However, this effect is not universal and depends on motivational relevance, stimulus characteristics, cognitive resources, and task demands [36,37,38]. To the contrary, in younger adults, enhanced processing of potentially threatening or behaviorally relevant information may support learning, decision-making, and adaptation to changing environments [39,40,41]. Thus, emotional object-location memory may provide a valuable framework for investigating how body–environment integration is reorganized across adulthood according to different adaptive priorities.

The relevance of these mechanisms extends beyond cognitive performance to subjective health and everyday functioning. HRQoL is a multidimensional construct encompassing physical functioning, vitality, emotional well-being, social participation, and perceived general health [42,43,44]. Consistent with the WHO model of Healthy Ageing, HRQoL reflects the ability to maintain meaningful activities and adapt to changes in intrinsic capacities and environmental demands. Increasing evidence suggests that subjective health emerges from the interaction of multiple cognitive, emotional, bodily, and environmental processes rather than from a single determinant. Multidimensional interoceptive awareness has been associated with perceived physical functioning, multisensory integration, and subjective health in older adults [18,45,46,47]. Similarly, spatial cognition and emotional memory contribute to adaptive behavior and psychological well-being across adulthood [48,49].

Despite growing evidence supporting the relevance of these domains, previous studies have largely examined interoceptive functioning, emotional processing, and spatial memory as separate mechanisms. Consequently, little is known about whether these processes jointly contribute to HRQoL or whether their relationship changes across adulthood. In particular, it remains unclear whether multidimensional interoceptive functioning and emotional object-location memory represent complementary components of a common adaptive system supporting health and autonomy, and whether aging modifies the way these components contribute to everyday functioning.

The present study investigated whether two complementary components of body–environment integration, multidimensional interoceptive functioning, and emotional object-location memory jointly contribute to HRQoL in younger (Y) and older adults (OA). Participants completed an Object Location Memory task in virtual reality designed to independently assess object recognition (What), spatial location memory (Where), and object-location binding (What + Where) using negative, neutral, and positive stimuli. Interoceptive functioning was assessed through the Multidimensional Assessment of Interoceptive Awareness (MAIA) [24,50] and the Body Perception Questionnaire (BPQ) [51,52], whereas HRQoL was evaluated using the Short Form Health Survey (SF-36) [42,53].

Based on previous evidence, we hypothesized that adaptive dimensions of interoceptive functioning would be positively associated with HRQoL and that emotional object-location memory would provide an additional contribution by capturing individual differences in the efficiency of body–environment integration. Furthermore, because aging is characterized by changes in associative memory, emotional processing, and interoceptive functioning, we expected the relationship between bodily regulation, emotional spatial memory, and perceived health to differ between younger and older adults.

## 2. Materials and Methods

### 2.1. Study Design and Participants

This study examined the associations between multidimensional interoceptive functioning, emotional object-location memory, and HRQoL in younger and older adults (see descriptive statistics in Supplementary Table S1 and correlation matrices for younger and older adults in Supplementary Tables S2 and S3, respectively). Eighty-one participants took part in the study on voluntary basis: 38 older adults (Mean age = 66 years, SD = 6.23; Mean years of education = 13.92, SD = 2.18; 18 males, 20 females) and 43 younger adults (Mean age = 27 years, SD = 4.73; Mean years of education = 14.62, SD = 2.04; 17 males, 26 females). The study was carried out at the Laboratory of Cognitive Science and Immersive Virtual Reality (CS-IVR) of the Department of Psychology, University of Campania “Luigi Vanvitelli” (Caserta, Italy). Participants were recruited from March 2025 to May 2026, and both younger and older participants were recruited through word of mouth. Informed consent was obtained from all participants. Recruitment and testing were in conformity with the requirements of the local Institutional Ethics Committee [36/2023]. Exclusion criteria comprised a history of neurological, psychiatric, or general medical conditions, as well as any medical disorder potentially affecting cognitive functioning (e.g., Alzheimer’s disease, multiple sclerosis, or Parkinson’s disease). Participants were excluded if they obtained a score lower than 26 on the Mini-Mental State Examination (MMSE) or lower than 15.5 on the Montreal Cognitive Assessment (MoCA). To assess these eligibility criteria, participants underwent a cognitive screening evaluating global cognitive functioning, executive functions, and reasoning. The MMSE, MoCA, and Cognitive Estimation Test (CET) were administered as screening measures to identify potential cognitive impairments in both younger and older adults. No participants were excluded on the basis of these measures. Mean MMSE scores were 29.74 (SD = 0.44) in younger adults and 28.84 (SD = 0.87) in older adults; mean MoCA scores were 28.27 (SD = 1.41) in younger adults and 26.21 (SD = 1.90) in older adults; and mean CET scores were 16.69 (SD = 4.05) and 15.52 (SD = 3.97), respectively [54,55,56,57,58,59,60,61]. Scores were interpreted according to normative data from Italian population samples [57,59,60,61].

The Toronto Alexithymia Scale (TAS-20) [62] was administered exclusively as a screening measure to identify potential participants with clinically significant alexithymia. No participants were excluded on this basis, as all obtained scores were within the non-alexithymic range (≤51).

Regarding the participant flow, all recruited participants met the inclusion criteria. No participants were excluded based on screening assessments, and no participants withdrew or dropped out during testing, resulting in a final completion rate of 100% across the entire experimental protocol.

Finally, a sensitivity analysis [63] was conducted to estimate the minimum detectable effect size for the regression analyses. Using the smaller age-group sample (*n* = 38 older adults), with power set at 0.80 and α = 0.05, the analysis indicated that an effect size of at least Cohen’s f = 1.09 could be detected, corresponding to R^2^ = 0.52. This estimate was used as a conservative reference for the two age groups and should not be interpreted as evidence of adequate power to detect small or moderate effects.

### 2.2. Experimental Setting and Stimuli

Participants completed the experimental protocol in a quiet room at the Laboratory of Cognitive Sciences and Virtual Reality of the University of Campania “Luigi Vanvitelli.” They wore a head-mounted display (Meta Oculus Quest 2; Meta Platforms Technologies, LLC, Menlo Park, CA, USA) to perform the Virtual Reality task, which was developed using Unity (version 2022.3.16f1) [64]. An immersive virtual reality adaptation of the Object Location Memory task [9,65] was used to assess participants’ ability to remember the spatial locations of objects within the environment. According to Postma et al. [10,65], this ability can be divided into three main components, corresponding to the three subtasks employed in the present study: (a) object processing, (b) spatial location processing, and (c) object-location binding. The three subtasks were always administered in the same order, with the object recognition task (What) presented first, followed by the spatial location task (Where), and finally What + Where. The order of the emotional valence blocks was pseudorandomized across participants: the neutral block was always presented first, whereas the positive and negative blocks were administered in a randomized order. Different stimuli were created according to their emotional valence, modeled in Blender (Version 4.2) [66], and subsequently placed on a table using SketchUp (version 2023) [67]. The virtual room containing the table and the objects was developed in Unity (version 2022.3.16f1). To validate the emotional valence of the stimuli, an online questionnaire was developed using Google Forms (Google LLC, Mountain View, CA, USA) and administered to an independent sample of 28 participants (13 younger adults and 15 older adults). Participants viewed each object and first categorized the conveyed emotional valence by choosing among four options: positive, negative, neutral, or other. Subsequently, they evaluated the emotional intensity of the objects (positivity, negativity, and neutrality) on seven-point Likert scales. They also rated perceived valence (“How unhappy or happy do you feel while viewing this image?”, 1 = very unhappy, 7 = very happy), arousal (“How calm or aroused do you feel while viewing this image?”, 1 = very calm, 7 = very aroused), ease of recognition, aesthetic pleasantness, and perceived familiarity (i.e., how familiar the object appeared regardless of whether they had previously encountered it). All experimental items, including neutral geometric shapes, underwent this validation. In older adults, positive and negative stimuli elicited comparable levels of arousal, which were significantly higher than those for neutral stimuli (positive: M = 5.58, SD = 0.17; negative: M = 5.86, SD = 0.43; neutral: M = 3.39, SD = 0.27). Valence ratings followed the intended gradation, with positive, neutral, and negative stimuli receiving progressively lower ratings (positive: M = 5.21, SD = 0.71; neutral: M = 3.53, SD = 0.45; negative: M = 2.69, SD = 0.60). Recognition and familiarity ratings were high across all categories (recognition: positive M = 6.28, SD = 0.20; neutral M = 5.98, SD = 0.34; negative M = 5.88, SD = 0.45; familiarity: positive M = 6.19, SD = 1.05; neutral M = 5.70, SD = 0.31; negative M = 5.21, SD = 0.65). Emotional intensity ratings for the target category confirmed strong category salience (positive: M = 5.49, SD = 0.56; neutral: M = 5.79, SD = 0.34; negative: M = 5.51, SD = 0.54). A consistent pattern was observed in younger adults, with comparable arousal for positive and negative stimuli and lower arousal for neutral stimuli (positive: M = 5.16, SD = 0.59; negative: M = 5.68, SD = 0.80; neutral: M = 2.91, SD = 0.29). Valence ratings aligned with the categories (positive: M = 5.38, SD = 0.76; neutral: M = 3.54, SD = 0.21; negative: M = 2.43, SD = 0.69). Recognition and familiarity remained high (recognition: positive M = 6.28, SD = 0.39; neutral M = 5.17, SD = 0.34; negative M = 5.87, SD = 0.43; familiarity: positive M = 6.19, SD = 0.35; neutral M = 4.87, SD = 0.33; negative M = 5.26, SD = 0.64), and target emotional intensity ratings were strong across all conditions (positive: M = 5.67, SD = 0.48; neutral: M = 5.05, SD = 0.24; negative: M = 5.37, SD = 0.64).

The stimuli were divided into three categories: (a) negative (electric saw, knife, scalpel, cleaver, axe, bomb, hatchet, handgun); (b) neutral (cone, pyramid, sphere, parallelepiped, cylinder, cube); and (c) positive (ball, teddy bear, pillow, pinwheel, doll, headphones, gift box, pizza box). To ensure that all objects were presented under identical conditions, A white support surface measuring 70 × 90 cm and 2 cm thick was used [68]. Gray square bases (10.5 × 10.5 cm) were placed on the table [68], and the objects were positioned arbitrarily while ensuring an approximately uniform distribution across the table surface [65]. For each subtask, three levels of memory load were created by presenting three objects in the first trial, four objects in the second trial, and five objects in the third trial. This procedure was applied to each emotional valence condition (negative, neutral, and positive).

### 2.3. Object Location Memory Task

**Figure 1:**
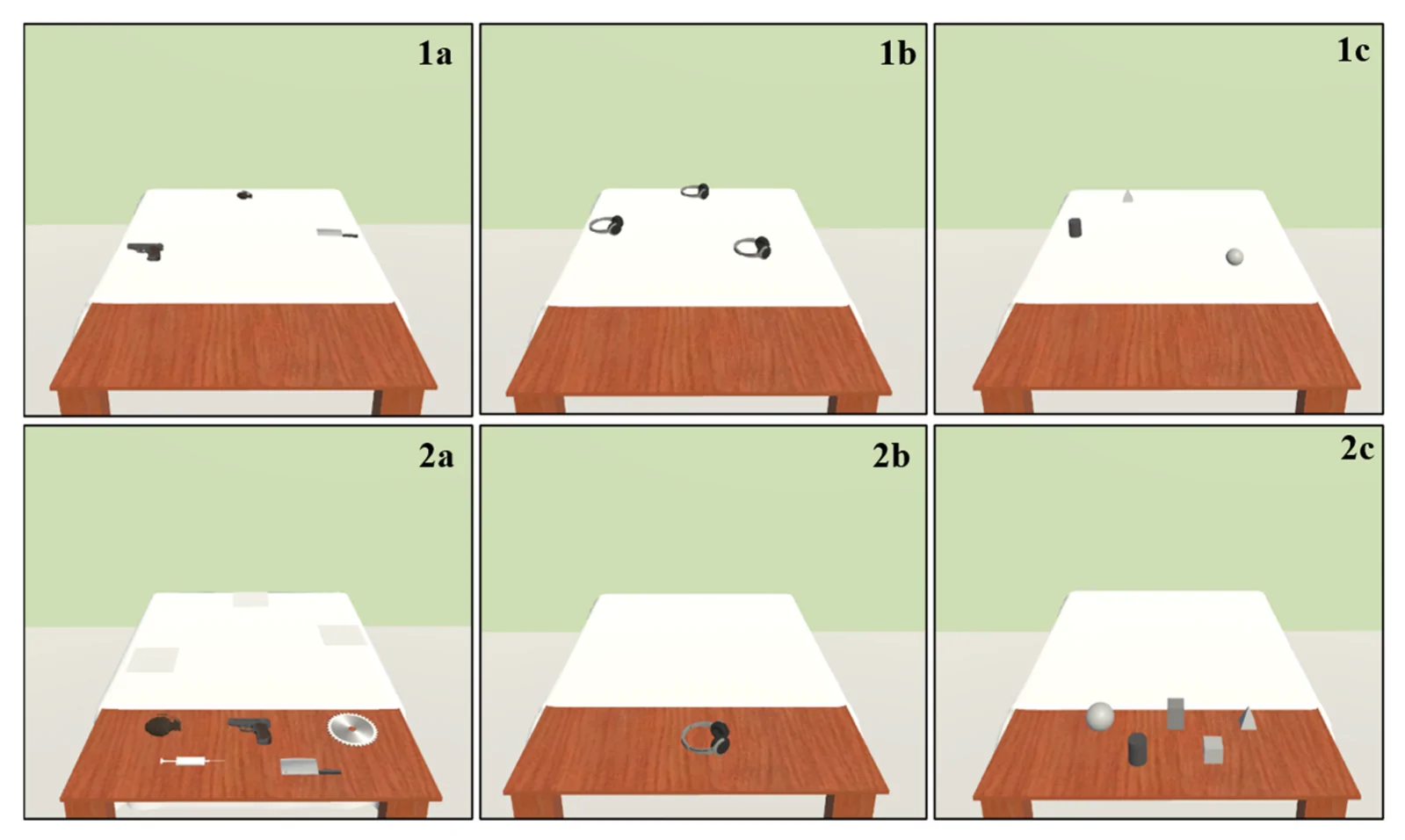
Examples of experimental conditions. The top row (panels (**1a**–**1c**)) illustrates the learning phase, and the bottom row (panels (**2a**–**2c**)) illustrates the corresponding testing phase. The left, middle, and right columns represent the What, Where, and What + Where tasks, respectively.

The Object Location Memory Task consisted of three subtasks corresponding to the three main processes involved in object-location memory: object processing (What), spatial-location processing (Where), and object-location binding (What + Where). In the What subtask, participants were required to remember the identity of the objects presented and to distinguish them from distractor objects (see Figure 1, panels 1a–2a). In the Where subtask, participants were required to remember the spatial locations previously occupied by the objects, while the same object was used for all locations (see Figure 1, panels 1b–2b). In the What + Where subtask, participants were required to remember both the identity of each object and its corresponding spatial location (see Figure 1, panels 1c–2c).

In all three subtasks, participants performed the task in an immersive virtual environment consisting of a neutral room. At the beginning of each trial, a configuration of objects arranged on a virtual table was presented for 5 s. After the encoding phase, the configuration disappeared, and participants were required to reconstruct the previously presented information using the joystick of the head-mounted display. No time limit was imposed for completing the reconstruction. Each trial ended when the participant informed the experimenter that the reconstruction was complete.

For each subtask and each emotional-valence condition (negative, neutral, and positive), participants completed three consecutive trials with progressively increasing memory load. The first trial included three objects, the second four objects, and the third five objects. Thus, each combination of subtask and emotional-valence condition comprised 12 total target responses across the three memory-load levels (3 + 4 + 5).

Performance was scored at the level of each individual target response. An invisible 5 × 5 cm grid was used on the virtual table to define the possible spatial locations of the objects. A binary accuracy score was assigned to each target (1 = correct, 0 = incorrect). In the What subtask, a response was scored as correct when the participant correctly reconstructed the identity of the required object. In the Where subtask, a response was scored as correct when the participant placed the object in the same grid cell that it had occupied during the encoding phase. In the What + Where subtask, a response was scored as correct only when both the object identity and its corresponding spatial location were correctly reconstructed. Thus, performance was assessed in terms of categorical accuracy rather than metric spatial error.

For each subtask and emotional-valence condition, performance across the three memory-load trials was aggregated at the item level. Specifically, the numbers of correct target responses obtained in the three-item, four-item, and five-item trials were combined, yielding a total of 12 target responses per condition. The composite score was then expressed as percentage accuracy, calculated as follows: Accuracy (%) = (total number of correct target responses/12) × 100. Accordingly, scores ranged from 0% to 100%, with higher percentages indicating better performance. The resulting scores represented object-recognition accuracy in the What condition, spatial-location memory accuracy in the Where condition, and object-location binding accuracy in the What + Where condition. These composite accuracy scores were subsequently used as spatial-memory predictors in the regression analyses.

The present task has not been subjected to a formal psychometric validation study. However, the same experimental paradigm has previously been applied in a single-case study involving a patient with a brain lesion [9]. This previous study provides evidence of prior experimental use of the paradigm but should not be considered a formal validation of the task.

### 2.4. Interoceptive Awareness Questionnaires and Daily Living Functioning

Interoceptive awareness was assessed using two self-report questionnaires: the Body Perception Questionnaire–Short Form (BPQ-22) [51,52] and the Multidimensional Assessment of Interoceptive Awareness (MAIA) [24,50]. Body Perception Questionnaire–Short Form (BPQ-22) [51,52]. The questionnaire comprises 22 items organized into three subscales: (a) Body Awareness (12 items), which measures awareness of general bodily sensations (e.g., tension, pain, and fatigue); (b) Subdiaphragmatic Reactivity (5 items), which assesses autonomic reactivity related to processes below the diaphragm (e.g., gastrointestinal sensations); and (c) Supradiaphragmatic Reactivity (5 items), which evaluates autonomic reactivity associated with processes above the diaphragm (e.g., respiratory and cardiac sensations). The Italian validation of the BPQ-22 reported good internal consistency, with Cronbach’s alpha coefficients ranging from 0.79 to 0.86 across the three subscales [51]. In the present sample, the internal consistency Cronbach’s alpha for the overall scale was 0.80.

The Multidimensional Assessment of Interoceptive Awareness (MAIA) [24,50]. It is a 32-item self-report measure that assesses several dimensions of interoceptive awareness. It consists of eight subscales: (a) Noticing (4 items), assessing awareness of comfortable, uncomfortable, and neutral bodily sensations; (b) Not-Distracting (3 items), evaluating the tendency to attend to rather than ignore sensations of pain or discomfort; (c) Not-Worrying (3 items), assessing the tendency not to experience emotional distress in response to unpleasant bodily sensations; (d) Attention Regulation (7 items), measuring the ability to sustain and control attention toward bodily sensations; (e) Emotional Awareness (5 items), assessing awareness of the relationship between bodily sensations and emotional states; (f) Self-Regulation (4 items), evaluating the ability to regulate distress by focusing attention on bodily sensations; (g) Body Listening (3 items), assessing the tendency to actively listen to bodily signals for insight; and (h) Trusting (3 items), evaluating the extent to which the body is experienced as safe and trustworthy. Previous validation studies have shown good internal consistency for the MAIA, with Cronbach’s alpha coefficients ranging from 0.66 to 0.87 across the eight subscales [24,52]. The MAIA (8 subscales) and BPQ (3 subscales) scales will be used as interoceptive predictors. In the present sample, the internal consistency Cronbach’s alpha for the overall scale was 81.

Thirty-six-Item Short Form Health Survey (SF-36) [53]. SF-36 is a 36-item self-report questionnaire designed to assess perceived health status across eight domains encompassing functional status, well-being, and overall health perception [69]. These domains include Physical Functioning, Role Limitations Due to Physical Health, Role Limitations Due to Personal or Emotional Problems, Energy/Fatigue, Emotional Well-Being, Social Functioning, Bodily Pain, and General Health. Each subscale is scored on a scale ranging from 0 to 100, with lower scores reflecting poorer perceived health and higher scores indicating better perceived health. The subscales can be interpreted independently to examine specific aspects of health and well-being. In the present study, analyses focused on the subscales primarily related to physical health: (a) Physical Functioning, assessing perceived physical functioning; (b) Role Limitations Due to Physical Health, evaluating the extent to which physical health interferes with everyday activities; (c) Emotional Well-Being, evaluating perceived emotional health, including positive and negative mood; (d) Role Limitations Due to Personal or Emotional Problems, assessing the extent to which emotional problems interfere with work and daily activities; (e) Energy/Fatigue, measuring perceived vitality and fatigue; (f) Social Functioning, assessing the extent to which physical or emotional health interferes with social activities; and (g) General Health, providing an overall evaluation of perceived health status. The SF-36 scales (8 subscales) will be used as outcomes.

### 2.5. Procedure and Experimental Design

**Figure 2:**
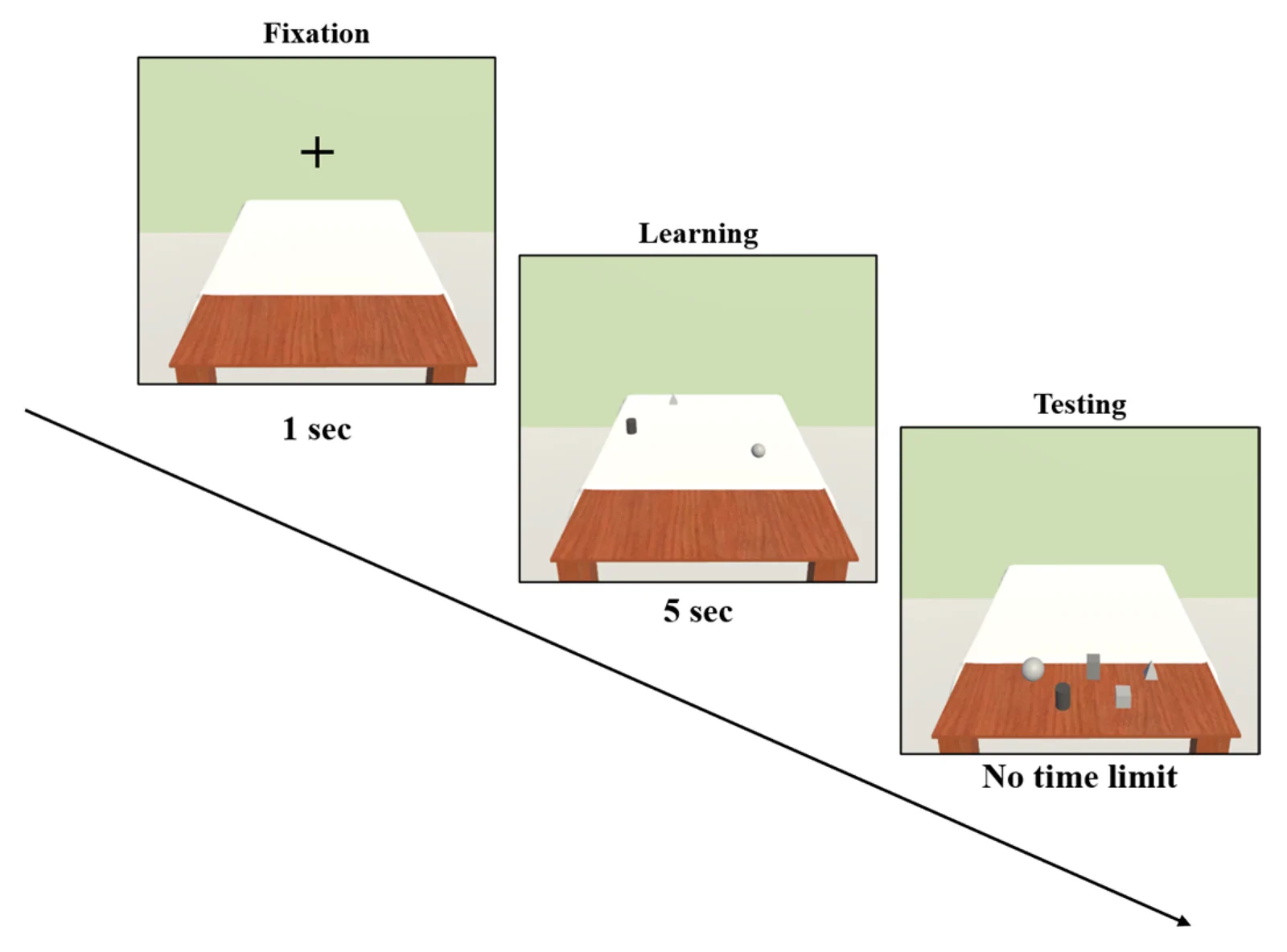
Example of the experimental flow for the What + Where Neutral condition. Participants were first presented with a fixation cross (starting from top left) for 1 s, followed by a triad of neutral objects presented for 5 s during the encoding phase. The objects were then removed, and the testing phase began, during which participants reconstructed the previously presented object–location configuration without a time limit.

After providing informed consent, participants underwent a neuropsychological assessment and completed self-report questionnaires assessing interoceptive awareness (MAIA and BPQ-22) and everyday functioning (SF-36). Subsequently, participants received both written and verbal instructions regarding the Object Location Memory Task (see Figure 2). Before the experimental session, participants completed a practice phase for each of the three subtasks using colored geometric cubes instead of the experimental objects. The experimental session began only after participants demonstrated an adequate understanding of the task requirements. Each subtask comprised three experimental blocks corresponding to the negative, neutral, and positive emotional valence conditions. The neutral condition was presented in an initial block, whereas the positive and negative conditions were pseudorandomized. Prior to each task, participants underwent a training session. First, participants signed the written informed consent form. They then completed the neuropsychological screening assessment (MMSE, MoCA, and CET) and the TAS-20, followed by the self-report questionnaires assessing interoceptive awareness (MAIA and BPQ-22) and health-related quality of life (SF-36). Subsequently, participants completed the familiarization training session before performing the experimental virtual reality task (comprising the What, Where, and What + Where subtasks). The entire experimental session lasted approximately 1 h.

### 2.6. Data Analysis

To examine the relationships between interoceptive measures, spatial measures, and self-reported health-related quality of life, the analyses were conducted in two sequential steps for each SF-36 domain and separately in the Young and Older Adult groups. Each SF-36 domain was treated as a continuous outcome.

In the first step, a bootstrap-based LASSO procedure was applied exclusively to the interoceptive predictors (BOA, SUP, BOASUP, and M1–M8) to identify interoceptive measures showing relatively stable selection across resampled datasets. LASSO is a regularized regression approach that combines coefficient shrinkage with variable selection by imposing an L1 penalty on the regression coefficients [70]. For each SF-36 outcome and age group, 1000 bootstrap samples were generated with replacement, and a Gaussian LASSO model was fitted within each bootstrap sample using cross-validation to select the regularization parameter corresponding to the minimum cross-validated mean squared error (λmin). The implementation was based on the “glmnet”(version 5.0) framework [71]. For each interoceptive predictor, selection frequency was calculated as the percentage of bootstrap samples in which the predictor retained a non-zero coefficient at λmin. Predictors selected in at least 50% of the bootstrap samples were retained for the second step. The use of repeated resampling to evaluate the robustness of variable-selection results is consistent with the general rationale underlying stability-oriented selection procedures [72].

In the second step, the interoceptive predictors retained from the first step were entered into a multiple linear regression model together with the complete set of prespecified spatial predictors (What Negative, What Neutral, What Positive, Where Negative, Where Neutral, Where Positive, What + Where Negative, What + Where Neutral, and What + Where Positive). Spatial predictors retained a priori in the second-stage models rather than subjected to variable selection, because the aim of this step was to examine the contribution of the interoceptive measures identified in the first step within a model that simultaneously accounted for the prespecified spatial measures. Thus, the two-stage procedure first reduced the interoceptive predictor set and subsequently evaluated the resulting interoceptive measures in combination with the full set of spatial predictors. In the LASSO implementation, this was achieved by assigning a penalty factor of zero to the spatial predictors, thereby preventing their coefficients from being penalized while retaining them in the model [71].

For each outcome and age group, the resulting second-stage model was estimated to use least-squares multiple linear regression. Model fit was summarized using R^2^, adjusted R^2^, the overall F-test, regression coefficients, standard errors, and associated *p*-values (See non-significant models in Supplementary Table S2). Ninety-five percent bootstrap confidence intervals for the regression coefficients were estimated from 5000 bootstrap resamples of the dataset [73]. These confidence intervals were calculated on the predictor set defined in the first step and therefore did not repeat the LASSO selection procedure.

The predictive performance of model was additionally assessed using leave-one-out cross-validation (LOOCV), with RMSE, MAE, and predictive R^2^ used to quantify out-of-sample prediction [74,75]. Finally, a permutation test based on 5000 random permutations of the outcome variable was performed for each final model to assess whether the observed overall model fit was greater than expected under a null distribution obtained by disrupting the correspondence between predictors and outcome. Permutation *p*-values were calculated using the standard +1 correction [76].

## 3. Results

For Physical Functioning (PF), predictor selection frequencies differed between groups. In the older adult group, M3 had the highest selection frequency (87.0%), followed by M1 (86.9%), M2 (66.5%), M4 (53.3%), and M8 (53.2%). The remaining interoceptive predictors were selected in fewer than 50% of bootstrap samples in both groups. In Young adults, no predictors were selected (<50%).

In the older adult group, the multiple regression analysis with Physical Functioning (PF; SF-36) as the criterion variable yielded a significant model, F(14, 23) = 3.715, *p* = 0.002, R^2^ = 0.693, adjusted R^2^ = 0.506. Noticing (M1), Not-Worrying (M3), What Negative, Where Negative, Where Neutral, and What + Where positive emerged as significant predictors of perceived physical functioning (Table 1).

Specifically, greater attention to bodily sensations was associated with lower perceived physical functioning, whereas the ability to maintain attention toward bodily signals without distraction and reduced emotional reactivity toward unpleasant bodily sensations were associated with higher Physical Functioning scores. Object-location memory predictors also showed emotion-dependent patterns. Performance in the What Negative and Where Negative conditions negatively predicted Physical Functioning, indicating that higher accuracy in processing negatively valenced object information and spatial locations was associated with lower perceived physical functioning. Conversely, Where Neutral and What + Where Positive performance positively predicted Physical Functioning. Together, these findings indicate that perceived physical functioning in older adults was related to selective dimensions of interoceptive regulation and emotionally modulated object-location memory. Finally, permutation testing indicated that the observed model fit was unlikely to be explained by chance (*p* = 0.001).

In the younger adult group, the multiple regression analysis with Physical Functioning (PF; SF-36) as the criterion variable did not yield a significant overall model (F < 1). LOOCV provided an out-of-sample assessment of these models. For Older adults, the model yielded an RMSE of 8.80, an MAE of 7.48, and a predictive R^2^ of 0.18, indicating that the model retained positive predictive explanatory value when evaluated on participants not used to fit the corresponding model. In young, RMSE was 7.19, MAE was 5.36, and predictive R^2^ was −0.05.

For Energy/Fatigue (EF), predictor selection frequencies differed between groups. In the older adult group, M8 had the highest selection frequency (74.5%), followed by M7 (73.1%) and M3 (69.9%). The remaining interoceptive predictors were selected in fewer than 50% of bootstrap samples in both groups. In Young adults, no predictors were selected (<50%).

In the older adult group, the multiple regression analysis with Energy/Fatigue (EF; SF-36) as the criterion variable yielded a significant model, F(12, 25) = 2.918, *p* = 0.01, R^2^ = 0.583, adjusted R^2^ = 0.383. Not-Worrying (M3), Where Negative and What + Where Negative emerged as significant predictors of perceived Energy/Fatigue (Table 2).

Specifically, higher levels of Not-Worrying (M3) were associated with higher perceived energy and vitality, indicating that reduced emotional concern in response to unpleasant sensations contributed to the prediction of perceived vitality. Conversely, What Negative and What + Where Negative performance negatively predicted Energy/Fatigue. Specifically, stronger object-location binding for negative stimuli was associated with lower perceived energy levels. Overall, the model suggests that vitality in older adults was primarily explained by adaptive interoceptive functioning, with an additional contribution of emotionally modulated object-location memory. Finally, permutation testing indicated that the observed model fit was unlikely to be explained by chance (*p* = 0.01).

In the younger adult group, the multiple regression analysis with Energy/Fatigue (EF; SF-36) as the criterion variable did not yield a significant overall model, F(9, 33) = 1.052, *p* = 0.42.

LOOCV provided an out-of-sample assessment of these models. For Older adults, the model yielded an RMSE of 12.90, an MAE of 10.59, and a predictive R^2^ of 0.10, indicating that the model retained positive predictive explanatory value when evaluated on participants not used to fit the corresponding model. In young, RMSE was 23.48, MAE was 18.74, and predictive R^2^ was −0.23.

For Emotional Well-Being (EWB), predictor selection frequencies differed between groups. In the older adult group, M1 had the highest selection frequency M7 (68.1%), followed by M3 (50.5%). In Young adults, BOA was most frequently selected (61.1%). The remaining interoceptive predictors were selected in fewer than 50% of bootstrap samples in both groups.

In the older adult group, the multiple regression analysis with Emotional Well-Being (EWB; SF-36) as the criterion variable yielded a significant model, F(13, 24) = 2.767, *p* = 0.01, R^2^ = 0.539, adjusted R^2^ = 0.344. Body Listening (M7), What + Where Negative emerged as significant predictors of perceived Emotional Well-Being (Table 3). Specifically, Body Listening (M7) was associated with better emotional well-being, indicating that greater ability to use bodily information contributed to the prediction of subjective emotional health. Object-location memory showed that What + Where Negative negatively predicted Emotional Well-Being. These findings suggest that emotional well-being in older adults was associated with both adaptive interoceptive functioning and selective processing of emotionally relevant spatial information. Permutation testing indicated that the observed model fit was unlikely to be explained by chance (*p* = 0.0004).

In the younger adult group, the multiple regression analysis with Energy/Fatigue (EF; SF-36) as the criterion variable did not yield a significant overall model, F(9, 33) = 1.052, *p* = 0.19.

LOOCV provided an out-of-sample assessment of these models. For Older adults, the model yielded an RMSE of 13.39, an MAE of 11.70, and a predictive R^2^ of 0.03, indicating that the model retained positive predictive explanatory value when evaluated on participants not used to fit the corresponding model. In young, RMSE was 10.61, MAE was 15.66, and predictive R^2^ was −0.21.

For Social Functioning (SF), predictor selection frequencies differed between groups. In the older adult group, M4 had the highest selection frequency (60.8%), followed by M3 (59.4%), M2 (59.2%), BOA (58.6%), and M8 (50.5%). In Young adults, SUP was most frequently selected (52.8%). The remaining interoceptive predictors were selected in fewer than 50% of bootstrap samples in both groups.

In the older adult group, the multiple regression analysis with Social Functioning (SF; SF-36) as the criterion variable yielded a significant model, F(14, 23) = 2.928, *p* = 0.01, R^2^ = 0.640, adjusted R^2^ = 0.421. Body Awareness/Perception (BOA), Not-Distracting (M2), and What + Where Neutral emerged as significant predictors of perceived Social Functioning (Table 4). Interoceptive predictors showed distinct patterns of contribution. Not-Distracting (M2) positively predicted Social Functioning, indicating that adaptive regulation, interpretation, and confidence in bodily sensations were associated with better perceived social functioning. Conversely, Body Awareness/Perception (BOA) showed negative associations with Social Functioning, suggesting that greater focus on bodily states was inversely related to perceived social functioning. Regarding object-location memory, What + Where Neutral performance negatively predicted Social Functioning. Thus, higher accuracy in binding object identity and spatial location for neutral stimuli was associated with lower social functioning scores. Overall, the model indicates that social functioning in older adults was associated with specific patterns of interoceptive regulation and object-location memory. Permutation testing indicated that the observed model fit was unlikely to be explained by chance (*p* = 0.01).

In the younger adult group, the multiple regression analysis with Energy/Fatigue (EF; SF-36) as the criterion variable did not yield a significant overall model, F(9, 33) = 1.052, *p* = 0.34. LOOCV provided an out-of-sample assessment of these models. For Older adults, the model yielded an RMSE of 15.72, an MAE of 11.84, and a predictive R^2^ of −0.03, indicating that the model retained negative predictive explanatory value when evaluated on participants not used to fit the corresponding model. In young, RMSE was 22.80, MAE was 11.84, and predictive R^2^ was −0.26.

For Pain (P), predictor selection frequencies differed between groups. In Older adults, M3 was most frequently selected (68.7%). In the Young adult group, BOA had a selection frequency (77.7%), followed by M2 (59.0%). The remaining interoceptive predictors were selected in fewer than 50% of bootstrap samples in both groups.

In the older adult group, the multiple regression analysis with Pain (P; SF-36) as the criterion variable did not yield a significant overall model, F(10, 27) = 1.052, *p* = 0.29.

In the young adult group, the multiple regression analysis with Pain (P; SF-36) as the criterion variable yielded a significant model, F(11, 31) = 3.160, *p* = 0.005, R^2^ = 0.528, adjusted R^2^ = 0.361. Body Awareness/Perception (BOA), Where Neutral, and What + Where positive emerged as significant predictors of perceived Pain (Table 5).

Body Awareness/Perception showed a negative association with Bodily Pain scores, indicating that greater focus on bodily sensations was associated with lower perceived pain-related functioning. Object-location memory showed different patterns depending on stimulus valence and processing component. Where Neutral performance positively predicted Bodily Pain scores, suggesting that more efficient processing of neutral spatial information was associated with better pain-related functioning. Conversely, What + Where Positive performance showed negative associations with Bodily Pain scores, indicating that stronger processing of positive spatial information was related to lower perceived pain-related functioning.

Overall, these findings indicate that pain-related functioning in younger adults is associated with specific aspects of bodily awareness and emotionally modulated spatial memory. While neutral spatial processing showed a positive relationship with pain-related functioning, enhanced processing of positive spatial information showed inverse associations.

## 4. Discussion

The present study investigated whether multidimensional interoceptive functioning and emotional object-location memory are associated with health-related quality of life across adulthood. By combining subjective measures of interoception (MAIA and BPQ) with an emotional Object Location Memory task performed in virtual reality, we examined whether individual differences in the regulation and interpretation of bodily sensations and in the processing of emotionally relevant environmental information were associated with different dimensions of perceived health.

Within each age group, specific dimensions of interoceptive functioning and emotional object-location memory were associated with selected HRQoL domains.

In older adults, Not-Worrying (M3) was positively associated with Physical Functioning and Energy/Fatigue, while Body Listening (M7) was positively associated with Emotional Well-Being. Instead, Not-Distracting (M2) was positively associated with Social Functioning. These findings suggest that the relationship between interoception and perceived health may depend less on the overall extent to which bodily sensations are noticed and more on how bodily information is attended to, regulated, and interpreted. In particular, the positive associations involving Not-Worrying, Not-Distracting, and Body Listening may be compatible with the possibility that reduced emotional concern in response to unpleasant bodily sensations, the ability to remain engaged with bodily information without excessive distraction, and the use of bodily information as a source of guidance are related to more favorable perceptions of health. This interpretation is consistent with multidimensional accounts of interoception, in which the detection of bodily signals, attentional regulation, emotional appraisal, and the interpretation of bodily information represent partially distinct processes rather than a single unitary construct [3,4,32]. Importantly, however, greater awareness of bodily sensations was not uniformly associated with more favorable HRQoL. Bodily Noticing (M1) was negatively associated with Physical Functioning, whereas BPQ-based Body Awareness/Perception (BOA) was negatively associated with Social Functioning. These findings further suggest that interoceptive functioning is multidimensional and that simply detecting or monitoring bodily sensations may not have the same functional significance as being able to regulate attention toward those sensations or respond to them without excessive concern. The negative association between Noticing and Physical Functioning may indicate that greater attention to bodily sensations is accompanied by lower perceived physical functioning. Similarly, the negative association between BOA and Social Functioning suggests that greater awareness of autonomic bodily states was associated with lower perceived social functioning.

Regarding older adults, our findings also showed that emotional object-location memory contributed to selected HRQoL outcomes, although the observed associations varied according to the emotional valence of the stimuli and the component of memory assessed. For Physical Functioning, better performance for negative object information (What Negative) and negative spatial information (Where Negative) was associated with lower perceived physical functioning, whereas better performance for neutral spatial information (Where Neutral) and positive object-location binding (What + Where Positive) was associated with higher Physical Functioning scores. For Energy/Fatigue, What Negative and What + Where Negative were negatively associated with perceived energy and vitality, indicating that stronger processing of negative object information and negative object-location associations was related to lower perceived energy. Similarly, What + Where Negative was negatively associated with Emotional Well-Being, while What + Where Neutral was negatively associated with Social Functioning.

Taken together, these findings indicate that the relationship between emotional object-location memory and HRQoL was not uniform but varied as a function of both emotional valence and the specific cognitive component assessed. In particular, associations involving negative information were predominantly negative, whereas positive and neutral information showed more differentiated associations across HRQoL domains.

This pattern can be considered in relation to previous research on age-related changes in emotional information processing and the positivity effect described within Socioemotional Selectivity Theory [33,35,38]. From this perspective, the emotional relevance assigned to environmental information may change across adulthood and may be reflected in differences in the processing of positive and negative information. However, the present findings do not directly demonstrate a positivity effect, nor do they establish that older adults preferentially process positive information or suppress negative information. Rather, they indicate that performance on specific positive, neutral, and negative object-location measures was differentially associated with subjective HRQoL. The negative associations between performance for negative information and several HRQoL domains may reflect the complex relationship between emotional information processing and subjective health, whereas the positive association between What + Where Positive and Physical Functioning may be compatible with the relevance of positive environmental information for perceived health.

Instead, in younger adults, better performance for positive object-location associations (What + Where Positive) and higher interoceptive awareness (BOA) were associated with selected pain-related dimensions, with stronger positive object-location binding and higher BOA scores being associated with lower perceived pain. Both measures also showed inverse associations with selected HRQoL domains. This pattern suggests that, in younger adults, more efficient encoding of positively valenced information and greater awareness of internal bodily states may be associated with individual differences in perceived pain. One possible interpretation is that the ability to efficiently encode positive environmental information and to monitor internal bodily states may characterize individuals who report lower levels of perceived pain. This interpretation is consistent with evidence that emotional valence can influence object-location memory and that interoceptive processes are involved in the perception and regulation of pain [77].

Overall, the results indicate that emotional valence may be relevant to the relationship between spatial memory and HRQoL.

Furthermore, an important feature of the present study was the simultaneous consideration of interoceptive functioning and emotional object-location memory. The findings suggest that both domains may be associated with HRQoL, although their contributions differed across HRQoL dimensions and age groups. This supports the usefulness of considering bodily and environmental information together rather than treating interoception and spatial-emotional memory as entirely independent domains.

### Limitations

Some limitations should be considered when interpreting the present findings. The relatively small sample size may have contributed to overfitting and unstable estimates; therefore, the relatively high explained variance observed in some models should be interpreted cautiously, particularly in light of adjusted R^2^ and cross-validation estimates. The study design further precludes conclusions about causality or developmental change, and the separate analyses of younger and older adults do not provide a direct statistical test of age-related differences in regression coefficients. Thus, differences in the observed patterns across age groups should not be interpreted as statistically established group differences (see Supplementary Table S5 for between-group comparisons of interoceptive and spatial variables). Moreover, the Object Location Memory task has not yet undergone formal psychometric validation. Although it has been previously used in a single-case study involving a patient with a brain lesion [9], this represents prior experimental use rather than formal validation.

Finally, body–environment integration should be considered a theoretical framework for interpreting the findings rather than a mechanism directly demonstrated by the present analyses. Alternative explanations, including differences in task engagement, individual emotional processing styles, or other unmeasured characteristics of the sample, cannot be excluded. Future longitudinal and experimental studies, ideally with larger samples and validated measures of emotional object-location memory, will be needed to assess the stability of these associations and clarify the mechanisms underlying the observed relationships.

## 5. Conclusions

The present study provides exploratory evidence that multidimensional interoceptive functioning and emotional object-location memory are associated with selected dimensions of health-related quality of life in younger and older adults. Our results showed that the pattern of associations varied between younger and older adults, suggesting that different dimensions of bodily and emotional-spatial processing may be relevant to perceived health across adulthood. These findings provide a basis for further investigation of the relationships between interoceptive functioning, emotional memory, and health-related quality of life.

Overall, the study contributes to an integrated perspective on body–environment relationships in healthy aging and provides a framework for future research examining how bodily and environmental information may jointly relate to perceived health and everyday functioning.

## Figures and Tables

**Table 1:** The table shows the effect of predictors that contributed significantly to the model in relation to Physical Functioning as a criterion in older adult group.

Variable	Estimate	Std_Error	t_Value	*p*_Value	CI_Lower	CI_Upper
(Intercept)	59.256	13.302	4.455	0.000 ***	31.740	86.773
M3	5.279	2.328	2.268	0.033 *	0.464	10.094
M1	−4.041	1.435	−2.816	0.010 *	−7.010	−1.072
M2	2.623	1.696	1.546	0.136	−0.886	6.133
M4	2.363	1.929	1.225	0.233	−1.628	6.353
M8	1.399	1.622	0.862	0.397	−1.957	4.755
What Negative	−0.157	0.078	−2.006	0.057 *	−0.318	0.005
What Neutral	0.070	0.109	0.642	0.527	−0.155	0.295
What Positive	0.096	0.087	1.093	0.286	−0.085	0.276
Where Negative	−0.178	0.075	−2.375	0.026 *	−0.332	−0.023
Where Neutral	0.239	0.076	3.162	0.004 **	0.083	0.396
Where Positive	−0.097	0.101	−0.968	0.343	−0.305	0.111
What + Where Negative	0.066	0.096	0.689	0.498	−0.132	0.264
What + Where Neutral	−0.206	0.148	−1.391	0.177	−0.512	0.100
What + Where Positive	0.190	0.086	2.197	0.038 *	0.011	0.369

Note. Std_Error = standard error; CI_lower and CI_upper = lower and upper bounds of the 95% confidence interval, respectively. * *p* < 0.05, ** *p* < 0.01, *** *p* < 0.001.

**Table 2:** The table shows the effect of predictors that contributed significantly to the model in relation to Energy/Fatigue as a criterion in older adult group.

Variable	Estimate	Std_Error	t_Value	*p*_Value	CI_Lower	CI_Upper
(Intercept)	27.501	14.191	1.938	0.064	−1.725	56.728
M8	3.678	2.236	1.645	0.112	−0.926	8.283
M7	4.187	2.102	1.992	0.057	−0.142	8.516
M3	7.581	2.937	2.581	0.016 *	1.532	13.630
What Negative	−0.275	0.115	−2.387	0.025 *	−0.512	−0.038
What Neutral	0.159	0.179	0.888	0.383	−0.210	0.527
What Positive	0.066	0.138	0.478	0.637	−0.218	0.350
Where Negative	−0.184	0.118	−1.560	0.131	−0.427	0.059
Where Neutral	0.080	0.114	0.699	0.491	−0.155	0.314
Where Positive	0.146	0.125	1.165	0.255	−0.112	0.403
What + Where Negative	−0.276	0.127	−2.166	0.040 *	−0.539	−0.014
What + Where Neutral	−0.007	0.221	−0.033	0.974	−0.462	0.448
What + Where Positive	0.087	0.133	0.657	0.517	−0.187	0.361

Note. Std_Error = standard error; CI_lower and CI_upper = lower and upper bounds of the 95% confidence interval, respectively. * *p* < 0.05.

**Table 3:** The table shows the effect of predictors that contributed significantly to the model in relation to Emotional Well-Being as a criterion in older adult group.

Variable	Estimate	Std_Error	t_Value	*p*_Value	CI_Lower	CI_Upper
(Intercept)	41.508	13.860	2.995	0.006 **	13.019	69.997
M7	5.625	1.807	3.113	0.004 **	1.911	9.339
M3	5.719	3.017	1.895	0.069	−0.483	11.921
What Negative	−0.142	0.118	−1.204	0.239	−0.384	0.100
What Neutral	0.089	0.177	0.501	0.621	−0.275	0.452
What Positive	0.034	0.137	0.251	0.804	−0.247	0.316
Where Negative	−0.010	0.118	−0.083	0.934	−0.253	0.233
Where Neutral	0.060	0.114	0.527	0.603	−0.174	0.294
Where Positive	0.192	0.126	1.519	0.141	−0.068	0.451
What + Where Negative	−0.354	0.131	−2.690	0.012 *	−0.624	−0.083
What + Where Neutral	−0.217	0.228	−0.955	0.349	−0.685	0.251
What + Where Positive	0.147	0.137	1.074	0.293	−0.135	0.429

Note. Std_Error = standard error; CI_lower and CI_upper = lower and upper bounds of the 95% confidence interval, respectively. * *p* < 0.05, ** *p* < 0.01.

**Table 4:** The table shows the effect of predictors that contributed significantly to the model in relation to Social Functioning as a criterion in older adult group.

Variable	Estimate	Std_Error	t_Value	*p*_Value	CI_Lower	CI_Upper
(Intercept)	50.761	20.891	2.430	0.023 *	7.545	93.978
M4	4.853	2.712	1.790	0.087	−0.756	10.463
M3	5.316	3.425	1.552	0.134	−1.769	12.401
M2	7.667	2.678	2.863	0.009 **	2.127	13.206
BOA	−1.480	0.601	−2.461	0.022 *	−2.724	−0.236
M8	4.181	2.627	1.592	0.125	−1.253	9.615
What Negative	−0.153	0.133	−1.147	0.263	−0.428	0.123
What Neutral	0.107	0.186	0.573	0.572	−0.278	0.491
What Positive	−0.012	0.150	−0.083	0.935	−0.322	0.297
Where Negative	0.147	0.129	1.133	0.269	−0.121	0.414
Where Neutral	−0.037	0.127	−0.294	0.772	−0.301	0.226
Where Positive	−0.142	0.154	−0.924	0.365	−0.461	0.176
What + Where Negative	−0.007	0.144	−0.049	0.961	−0.305	0.290
What + Where Neutral	−0.649	0.255	−2.545	0.018 *	−1.177	−0.122
What + Where Positive	0.115	0.147	0.785	0.440	−0.188	0.418

Note. Std_Error = standard error; CI_lower and CI_upper = lower and upper bounds of the 95% confidence interval, respectively. * *p* < 0.05, ** *p* < 0.01.

**Table 5:** The table shows the effect of predictors that contributed significantly to the model in relation to Pain as a criterion in Young group.

Variable	Estimate	Std_Error	t_Value	*p*_Value	CI_Lower	CI_Upper
(Intercept)	133.098	25.066	5.310	0.000 ***	81.975	184.221
BOA	−2.121	0.594	−3.569	0.001 ***	−3.334	−0.909
M2	−3.073	2.106	−1.459	0.155	−7.368	1.222
What Negative	−0.018	0.124	−0.145	0.886	−0.270	0.234
What Neutral	0.161	0.158	1.019	0.316	−0.161	0.484
What Positive	−0.268	0.139	−1.930	0.063	−0.552	0.015
Where Negative	−0.188	0.130	−1.449	0.157	−0.453	0.077
Where Neutral	0.515	0.165	3.123	0.004 **	0.179	0.852
Where Positive	−0.019	0.152	−0.123	0.903	−0.329	0.292
What + Where Negative	−0.160	0.107	−1.501	0.143	−0.378	0.058
What + Where Neutral	−0.180	0.105	−1.723	0.095	−0.394	0.033
What + Where Positive	−0.209	0.101	−2.062	0.048 *	−0.416	−0.002

Note. Std_Error = standard error; CI_lower and CI_upper = lower and upper bounds of the 95% confidence interval, respectively. * *p* < 0.05, ** *p* < 0.01, *** *p* < 0.001.

## Data Availability

The datasets generated and/or analysed during the current study are not publicly available due to privacy and confidentiality restrictions regarding participant data but are available from the authors.

## Supplementary Materials

The following supporting information can be downloaded at: https://www.mdpi.com/article/10.3390/healthcare14203414/s1, Table S1: Descriptive Analyses; Table S2: Correlation matrix for young group; Table S3: Correlation matrix for Older Adults group; Table S4: General Health; Table S5: Role Limitations Due to Physical Health. Table S6: Role Limitations Due to Emotional Health. Table S7: T-test analysis.

## Abbreviations

The following abbreviations are used in this manuscript:

HRQoL	Health-related quality of life
Y	Younger
OA	Older adults
