# Supplementary material for: When the Body Meets Space: The Contribution of Interoception and Emotional Object-Location Memory as Predictors of Health-Related Quality of Life in Aging
Source: Healthcare (Basel). 2026 Oct 13;14(20):3414. doi: 10.3390/healthcare14203414 (PMC13704354; doi:10.3390/healthcare14203414)
Supplement: Supplementary file 1 [file healthcare-14-03414-s001.zip › healthcare-4519699-supplementary.pdf]

Supplementary Materials

Table S1. Descriptive Analyses.

Group	Variables	N	Mean	SD	Median	IQR	Q1	Q3	Min	Max	Skewness	Kurtosis
Young	PF	43	95.70	5.73	100.00	5.00	95.00	100.00	80.00	100.00	−1.332	3.900
	EF	43	48.84	21.40	50.00	30.00	35.00	65.00	0.00	95.00	−0.072	2.802
	EWB	43	58.60	17.08	60.00	24.00	48.00	72.00	24.00	84.00	−0.150	2.095
	SF	43	69.36	20.55	72.50	12.50	62.50	75.00	25.00	100.00	−0.326	2.921
	LRDPE	43	53.49	39.93	33.33	66.67	33.33	100.00	0.00	100.00	−0.040	1.478
	LRDSF	43	79.65	26.31	100.00	25.00	75.00	100.00	0.00	100.00	−1.245	3.799
	PF	43	87.90	14.60	90.00	22.50	77.50	100.00	45.00	100.00	−1.168	3.582
	GH	43	56.00	16.03	57.50	15.42	51.25	66.67	20.83	95.00	−0.270	3.112
	BOA	43	13.56	3.76	13.00	5.00	11.00	16.00	7.00	21.00	0.314	2.282
	SUP	43	9.74	1.75	10.00	2.00	8.00	10.00	8.00	14.00	1.076	3.517
	BOASUP	43	13.16	2.91	13.00	4.00	11.00	15.00	7.00	20.00	−0.017	2.441
	M1	43	3.20	1.18	3.50	1.88	2.13	4.00	0.75	5.00	−0.571	2.338
	M2	43	2.11	1.02	2.00	1.17	1.50	2.67	0.00	4.00	−0.161	2.313
	M3	43	2.78	0.99	3.00	1.17	2.33	3.50	0.67	4.33	−0.403	2.216
	M4	43	3.20	0.89	3.29	1.21	2.64	3.86	1.00	4.71	−0.294	2.707
	M5	43	3.61	1.00	3.80	0.80	3.30	4.10	1.00	5.00	−0.783	3.107
	M6	43	2.84	1.22	3.00	1.63	2.13	3.75	0.50	5.00	−0.158	2.327
	M7	43	2.89	1.14	3.00	1.67	2.00	3.67	0.33	5.00	−0.138	2.492
	M8	43	3.36	1.31	3.33	1.50	2.67	4.17	0.33	5.00	−0.629	2.738
	What Negative	43	71.69	19.32	71.11	32.50	54.17	86.67	26.11	100.00	−0.097	2.052
	What Neutral	43	65.63	16.89	68.33	22.78	55.56	78.33	27.78	100.00	−0.326	2.632
	What Positive	43	72.53	20.33	73.33	33.33	56.67	90.00	26.11	100.00	−0.310	2.114
	Where Negative	43	72.87	27.35	75.00	50.56	49.44	100.00	13.33	100.00	−0.455	1.909

	Where Neutral	43	82.60	18.67	91.67	30.83	69.17	100.00	35.00	100.00	−0.800	2.530
	Where Positive	43	82.20	20.47	91.67	36.67	63.33	100.00	37.22	100.00	−0.693	1.981
	What + Where Negative	43	49.14	20.34	50.00	29.44	35.83	65.28	0.58	86.67	−0.075	2.371
	What + Where Neutral	43	47.73	25.73	47.78	37.78	27.78	65.56	0.00	100.00	0.227	2.402
	What + Where Positive	43	50.70	25.36	53.33	34.44	32.22	66.67	0.00	100.00	0.060	2.274
Older Adults	PF	38	86.10	9.87	85.81	15.00	80.00	95.00	65.00	100.00	−0.289	2.315
	EF	38	66.24	13.77	65.50	22.50	56.25	78.75	35.00	95.00	−0.010	2.393
	EWB	38	72.89	13.78	72.00	19.00	65.00	84.00	40.00	96.00	−0.549	3.016
	SF	38	73.41	15.65	72.50	24.38	62.50	86.88	37.50	100.00	−0.165	2.555
	LRDPE	38	78.95	35.87	100.00	33.33	66.67	100.00	0.00	100.00	−1.434	3.490
	LRDSF	38	80.26	32.96	100.00	25.00	75.00	100.00	0.00	100.00	−1.470	3.715
	PF	38	77.91	18.52	77.50	30.00	67.50	97.50	35.00	100.00	−0.451	2.364
	GH	38	57.42	9.81	56.25	11.04	50.83	61.88	35.00	75.00	0.039	2.935
	BOA	38	13.34	3.56	13.00	5.00	11.00	16.00	7.00	21.00	0.477	2.616
	SUP	38	10.28	2.30	10.00	3.75	8.25	12.00	8.00	17.00	1.091	3.617
	BOASUP	38	13.76	3.22	14.00	3.00	12.00	15.00	7.00	21.00	0.172	3.213
	M1	38	2.57	1.21	2.50	1.69	1.81	3.50	0.00	5.00	−0.167	2.449
	M2	38	1.94	0.92	1.83	1.33	1.33	2.67	0.00	3.67	0.130	2.255
	M3	38	2.54	0.67	2.44	1.00	2.00	3.00	1.33	4.00	0.268	2.332
	M4	38	2.84	1.04	2.71	1.39	2.18	3.57	0.75	5.00	0.003	2.595
	M5	38	3.66	0.79	3.60	1.10	3.10	4.20	1.80	5.00	−0.195	2.600
	M6	38	3.09	0.90	3.05	1.19	2.56	3.75	1.00	5.00	−0.278	2.667
	M7	38	2.75	1.15	2.67	1.33	2.00	3.33	0.00	5.00	−0.330	3.199
	M8	38	3.83	1.00	4.00	1.33	3.33	4.67	1.33	5.00	−0.740	2.827
	What Negative	38	61.37	18.42	63.33	24.31	49.03	73.33	17.78	100.00	−0.329	2.826
	What Neutral	38	56.34	11.86	56.67	15.42	49.58	65.00	32.78	78.33	−0.325	2.354
	What Positive	38	68.56	17.73	71.11	17.92	61.67	79.58	26.11	100.00	−0.578	3.054

	Where Negative	38	49.20	22.33	45.56	34.72	31.94	66.67	17.78	100.00	0.502	2.338
	Where Neutral	38	58.85	19.35	59.72	20.97	48.75	69.72	15.00	100.00	−0.076	2.747
	Where Positive	38	56.17	19.65	57.78	26.11	38.89	65.00	26.11	91.67	0.169	2.086
	What + Where Negative	38	35.77	16.67	35.83	19.86	25.28	45.14	0.00	63.33	−0.073	2.372
	What + Where Neutral	38	20.00	11.58	19.72	13.61	13.75	27.36	0.00	41.67	0.043	2.296
	What + Where Positive	38	40.44	17.31	43.33	22.08	30.14	52.22	0.00	71.67	−0.738	2.979

Table S2. Correlation matrix for young group.

Spatial	BOA	SUP	B-S	M1	M2	M3	M4	M5	M6	M7	M8
What Negative	−0.08	−0.23	−0.25	−0.19	0.02	0.44	−0.02	−0.11	−0.03	−0.24	0.14
What Neutral	0.02	−0.22	−0.27	−0.17	−0.18	0.46	−0.01	−0.17	−0.06	−0.09	0.19
What Positive	−0.05	−0.13	−0.31	−0.26	−0.15	0.34	0.03	−0.19	−0.18	−0.36	−0.02
Where Negative	−0.26	0.25	0.14	0.27	0.05	−0.06	−0.05	0.21	0.07	0.15	−0.22
Where Neutral	−0.22	0.29	0.21	0.30	0.13	−0.20	0.00	0.13	0.16	0.35	−0.15
Where Positive	−0.28	0.18	0.20	0.10	0.19	−0.09	−0.19	0.02	0.08	0.22	−0.09
What + Where Negative	−0.39	0.08	−0.18	−0.06	0.22	−0.04	0.14	−0.05	−0.12	−0.04	−0.05
What + Where Neutral	−0.12	0.25	0.20	0.11	0.06	−0.31	−0.02	0.23	0.13	0.12	−0.17
What + Where Positive	−0.29	0.10	0.15	0.09	0.29	−0.10	−0.10	0.02	−0.01	0.21	−0.15

Table S3. Correlation matrix for Older Adults group.

Spatial	BOA	SUP	B-S	M1	M2	M3	M4	M5	M6	M7	M8
What Negative	0.12	0.10	0.10	0.08	−0.09	0.04	0.21	0.20	0.06	0.00	0.00
What Neutral	0.01	−0.09	−0.02	−0.02	0.05	0.29	0.09	0.04	−0.05	0.24	−0.10
What Positive	−0.01	0.15	0.34	0.07	−0.14	0.21	−0.07	−0.12	−0.24	−0.12	0.04
Where Negative	−0.03	−0.21	−0.08	−0.26	0.16	0.17	−0.01	−0.16	−0.04	0.29	0.07
Where Neutral	−0.08	−0.04	0.11	−0.02	−0.08	−0.06	−0.05	0.15	0.06	0.08	0.23
Where Positive	−0.22	−0.05	−0.14	−0.19	−0.24	0.01	0.28	0.04	0.13	−0.01	0.13
What +Where Negative	−0.15	0.15	0.14	0.18	−0.10	0.21	−0.03	0.08	−0.04	0.10	0.14
What + Where Neutral	0.00	−0.01	0.07	−0.01	0.29	0.26	0.04	−0.12	−0.09	0.17	−0.01
What + Where Positive	−0.14	0.05	−0.12	0.04	0.09	0.10	0.06	0.01	0.09	0.21	0.07

Non-significant models

Table S4. General Health.

Group	N	R^2	Adjusted_R^2	F	df1	df2	*p*
Young	43	0.170	−0.056	0.754	9	33	0.658
Older Adults	38	0.407	0.156	1.623	11	26	0.150

Table S5. Role Limitations Due to Physical Health.

Group	N	R^2	Adjusted_R^2	F	df1	df2	*p*
Young	43	0.311	0.123	1.652	9	33	0.141

Older Adults	38	0.102	−0.187	0.353	9	28	0.948

Table S6. Role Limitations Due to Emotional Health.

Group	N	R^2	Adjusted_R^2	F	df1	df2	*p*
Young	43	0.346	0.142	1.695	10	32	0.125
Older Adults	38	0.261	−0.012	0.956	10	27	0.502

T-TEST Analysis

Table S7. T-test analysis.

Variables	N_Y	Mean_Y	SD_Y	N_OA	Mean_OA	SD_OA	Mean_Diff.	t	df	*p*	CI_Lower	CI_Upper	p_FDR
BOA	43	13.56	3.76	38	13.34	3.56	0.22	0.27	78.62	0.79	−1.40	1.84	0.79
SUP	43	9.74	1.75	38	10.28	2.30	−0.53	−1.16	68.68	0.25	−1.45	0.38	0.38
BOASUP	43	13.16	2.91	38	13.76	3.22	−0.60	−0.88	75.22	0.38	−1.97	0.76	0.48
M1	43	3.20	1.18	38	2.57	1.21	0.63	2.37	77.35	0.02	0.10	1.16	0.05
M2	43	2.11	1.02	38	1.94	0.92	0.17	0.77	78.97	0.44	−0.26	0.59	0.52
M3	43	2.78	0.99	38	2.54	0.67	0.23	1.26	74.07	0.21	−0.14	0.61	0.35
M4	43	3.20	0.89	38	2.84	1.04	0.37	1.69	73.28	0.09	−0.07	0.80	0.17
M5	43	3.61	1.00	38	3.66	0.79	−0.06	−0.28	78.00	0.78	−0.45	0.34	0.79
M6	43	2.84	1.22	38	3.09	0.90	−0.25	−1.06	76.59	0.29	−0.72	0.22	0.41
M7	43	2.89	1.14	38	2.75	1.15	0.15	0.57	77.47	0.57	−0.36	0.65	0.63
M8	43	3.36	1.31	38	3.83	1.00	−0.47	−1.83	77.44	0.07	−0.98	0.04	0.14
What Negative	43	71.69	19.32	38	61.37	18.42	10.33	2.46	78.53	0.02	1.97	18.68	0.05
What Neutral	43	65.63	16.89	38	56.34	11.86	9.30	2.89	75.32	0.00	2.89	15.70	0.02
What Positive	43	72.53	20.33	38	68.56	17.73	3.97	0.94	78.99	0.35	−4.45	12.39	0.47
Where Negative	43	72.87	27.35	38	49.20	22.33	23.67	4.29	78.53	0.00	12.68	34.67	0.00
Where Neutral	43	82.60	18.67	38	58.85	19.35	23.75	5.60	77.00	0.00	15.31	32.19	0.00
Where Positive	43	82.20	20.47	38	56.17	19.65	26.03	5.83	78.44	0.00	17.15	34.91	0.00
What+Where Negative	43	49.14	20.34	38	35.77	16.67	13.36	3.25	78.58	0.00	5.17	21.55	0.01
What+Where Neutral	43	47.73	25.73	38	20.00	11.58	27.73	6.37	59.88	0.00	19.02	36.43	0.00
What+Where Positive	43	50.70	25.36	38	40.44	17.31	10.26	2.15	74.47	0.04	0.74	19.78	0.08
